# A Prospective Randomized Trial to Assess the Antireflux Effect of Antireflux Mucosectomy in the Porcine Model

**DOI:** 10.1155/2019/3286738

**Published:** 2019-02-24

**Authors:** Xuan Li, Weifeng Zhang, Meihong Chen, Shuchun Wei, Xiangyang Zhao, Guoxin Zhang

**Affiliations:** ^1^Department of Gastroenterology, The First Affiliated Hospital of Nanjing Medical University, Nanjing 210000, China; ^2^Department of Gastroenterology, The First School of Clinical Medicine of Nanjing Medical University, Nanjing 210000, China; ^3^Department of Gastroenterology, Nanjing Lishui People's Hospital, Lishui, Nanjing 211200, China; ^4^Department of Gastroenterology, Shengze Hospital, Suzhou 210000, China

## Abstract

**Background:**

Both long-term proton pump inhibitor use and surgical fundoplication have potential drawbacks as treatments for chronic gastroesophageal reflux disease (GERD). Our aim was to investigate the potential efficacy of antireflux mucosectomy (ARMS) in porcine and determine the optimal circumference of resection in relation to gastroesophageal junction (GEJ).

**Methods:**

Nine pigs were allocated into the following 3 groups by computerized randomization: group A: control, group B: 1/3 circumference of the esophagus, and group C: 2/3 circumference of the esophagus. We performed mucosectomy with a crescentic mucosal resection at 3 cm above the GEJ and 1 cm below the GEJ. The animals were kept on a liquid diet for 24 h prior to endoscopy. At 6 weeks, animals underwent esophagoscopy, barium radiography, gastric yield pressure (GYP), and gastric yield volume (GYV) determination.

**Results:**

The weight of swines has no significant difference, and all pigs had maintained their weight after the procedure. We both found scar formation at the GEJ in group B and C. Compared with group A and B, group C produced significantly higher GYP (24.23 ± 3.42 mmHg, *p* = 0.004) and significantly smaller GYV (2200.0 ± 238.96 mL, *p* = 0.028) after 6 weeks. Barium radiography showed that the width of the cardia was narrower (13.73 ± 1.19 mm, *p* = 0.032) in group C after 6-week postprocedure.

**Conclusion:**

Our study demonstrated the potential antireflux effect of ARMS. We also recommend the 2/3 circumference resection of mucosa at 3 cm distance from the GEJ.

## 1. Introduction

Gastroesophageal reflux disease (GERD) is a neuromuscular disorder with abnormal reflux of gastric contents into the esophagus [1]. It is a common disease in which mechanisms such as poor esophageal clearance, delayed gastric emptying, and low esophageal sphincter (LES) dysfunction, as a result of esophagogastric motility disorder [2]. The most common symptoms are heartburn, dysphagia, and regurgitation [3]. The other extraesophageal manifestations include chest pain [4], chronic hoarseness [5], and asthma [6].

The use of proton pump inhibitors (PPI) is the primary treatment of GERD, but in general, the effectiveness of PPI was limited. PPI provided an entirely symptomatic relief in 70 to 80% of patients [7, 8], so high dose of PPI and other ancillary medications seems to be effective in controlling the symptoms of GERD [9, 10]. Recent evidence has shown that chronic PPI therapy was related to defects in bone fracture, infectious complications, and absorption of vitamins and minerals [11–13]. Antireflux surgery is the most effective therapy for prompting the symptom relief of GERD. Concerns about the problematic side effects of antireflux surgery include flatulence, diarrhea, and bloating [14, 15]. As for the invasiveness of surgery, many endoscopic treatments have been developed as an alternative therapy, such as endoscopic anterior fundoplication, transoral incisionless fundoplication (TIF). However, these endoscopic devices have not yet withstood the test of clinical trials.

Recently, Inoue et al. reported antireflux mucosectomy (ARMS) was available for the treatment of GERD [16]. However, they did not evaluate which the area of ARMS produces the best results. We conducted this study to assess the potential efficacy of ARMS and determine the optimal circumference of resection in relation to gastroesophageal junction (GEJ) [17].

## 2. Methods

### 2.1. Study Design and Procedure of ARMS

**Figure 1:**
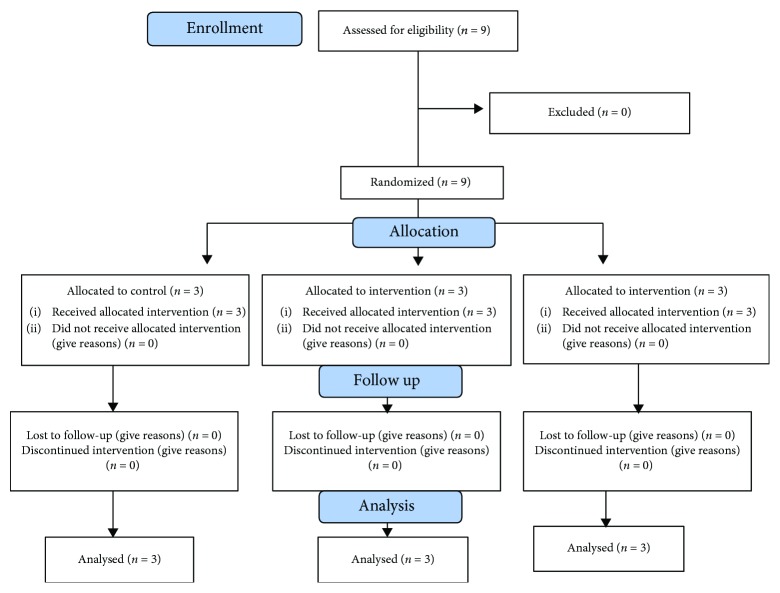
Flow diagram.

As shown as Figure 1, nine swines were allocated into the following 3 groups by computerized randomization: group A: control, group B: 1/3 circumference of the esophagus, and group C: 2/3 circumference of the esophagus. These pigs of the mixed breed were used in the study. They were kept on a liquid diet for 24 h prior to endoscopy. The study was approved by the Medical Ethics Committee of the First Affiliated Hospital of Nanjing Medical University.

The procedure was performed under conscious sedation. Midazolam, propofol, or both were administered to achieve deep sedation. Before ARMS, they underwent barium radiography to measure the width of the cardia and the time of wave. ARMS was performed by the use of a GIF-Q290 J (Olympus) with a transparent hood (D-2201-11304; Olympus) attached to the tip of the gastroscopy. For the procedures, an electrosurgical knife (KD-640L; Olympus) was used. STESD procedures were conducted by an experienced endoscopist with at least 5 years of experience in performing therapeutic gastrointestinal endoscopy.

**Figure 2:**
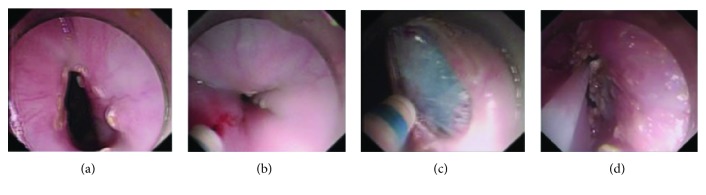
Procedures of ARMS: (a) marking; (b) submucosal injection; (c) mucosal resection; (d) submucosal excision; Figure 2 is reproduced from Li et al. [17] (under the Creative Commons Attribution License/public domain).

We performed mucosectomy with a crescentic mucosal resection at 3 cm above the GEJ and 1 cm below the GEJ with standardized techniques of endoscopic piecemeal mucosal resection (EPMR); the great curve of the gastric cardia was preserved. First, esophagus and stomach cavity were washed by normal saline if food residues were found. Second, we marked the mucosa along the margin of mucosal resection. Third is the submucosal injection of methylene blue solution. Fourth, a crescentic mucosal resection was performed at 3 cm above the GEJ and 1 cm below the GEJ. Finally, hemostasis was achieved using electrocoagulation if necessary (Figure 2).

### 2.2. Postprocedural Management and Outcome Measurement

All animals were kept on a liquid diet for 24 h subsequent to endoscopy. At 3 and 6 weeks, esophagoscopy and barium radiography were performed by another examiner who was blind to the experiment group. At 6 weeks, we sacrificed animals for gastric yield pressure (GYP) and gastric yield volume (GYV) determination.

The primary outcomes for this study were GYP and GYV. To determine GYP and GYV, a manometry catheter was placed into the stomach lumen, which was connected to a pressure transducer (solar 8000I, GE). The gastric outlet was ligated at the pylorus, and the stomach lumen was filled with normal saline by the use of the manometry catheter irrigation port (100 mL/min). The GYP was defined as intragastric pressure until reflux of irrigation fluid was noted in the esophagus. If the pressure led to a rupture of the specimen, this burst pressure threshold was noted as GYP. The GYV was defined as the total amount of infused water to the position of reflux detection. The secondary outcomes were the width of the cardia and the incidence of complications. The width of the cardia was measured by barium radiography.

### 2.3. Statistical Analysis

All statistical analyses were conducted with SPSS version 21.0 (SPSS Inc., Chicago, IL, USA). For this statistical analysis, the mean ± standard deviation was used for the continuous variables. For continuous variables, group comparisons were calculated by ANOVA.

## 3. Results

### 3.1. The Clinical Characteristics of Animals

A total of nine pigs were enrolled in our study; 3 procedures per each group were successfully performed. All procedures were completed satisfactorily, and all pigs were tolerated ARMS well without complications. Clinical characteristics of the included animals have been presented in Table 1. Most of the animals were male (*n* = 4, 57.1%). The mean weight in each group was as follows: group A: 35.17 ± 0.76 kilogram (kg); group B: 33.00 ± 3.61 kg; group C: 35.5 ± 1.80 kg. There was no significant difference between the 3 groups. The procedure time was longer in group C (78.67 ± 6.51 min, *p* = 0.006). In the postoperative course, no procedure-related complications or adverse events occurred.

### 3.2. Gastric Yield Pressure (GYP) and Gastric Yield Volume (GYV)

The baseline GYP of all pigs was 0. The mean GYP in each group was as follows: GYP in group B and C was higher than the control group. The mean GYV in each group was as follows: 2/3 circumference of esophagus mucosal resection could lead to obviously decrease the GYV (Table 1).

### 3.3. The Width of the Cardia

**Figure 3:**
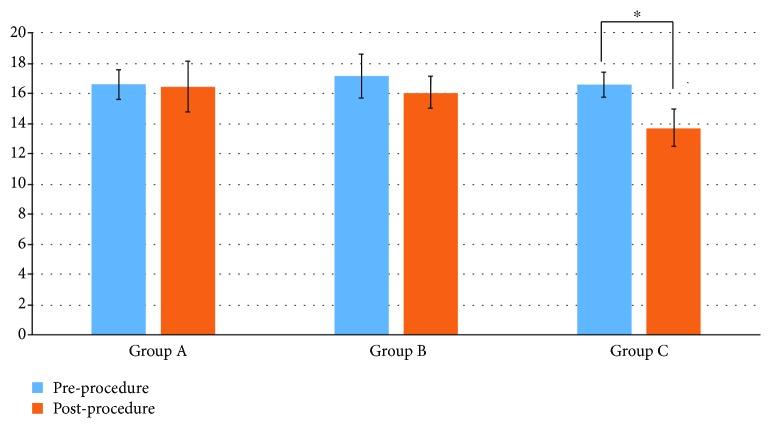
The result of the width cardia pre- and postprocedure.

The width of the cardia in each group after the ARMS was as follows: group A: 16.40 ± 1.17 mm; group B: 16.1 ± 1.06 mm; group C: 13.73 ± 1.19 mm. The results showed that significant differences were shown in group C at 6 weeks after the procedure (Figure 3).

**Figure 4:**
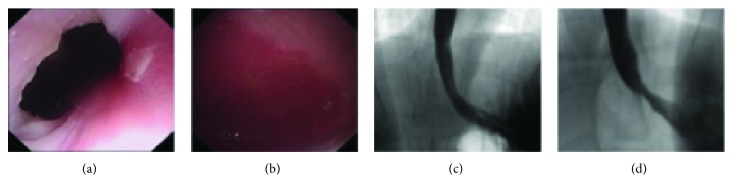
The result of endoscopy and barium radiography in group C: (a) esophageal mucosa at 6 weeks after ARMS; (b) stomach mucosa at 6 weeks after ARMS; (c) barium radiography before ARMS; (d) barium radiography after ARMS.

At 6-week follow-up, none of the 9 swines showed adverse events. Follow-up endoscopy and barium radiography showed that the width of the cardia had decreased significantly and scar formation occurred (Figure 4). The swines showed a significant improvement in the GYP and GYV. Gastroesophageal flap valve (GEFV) was II grade.

## 4. Discussion

This study showed that ARMS may be a possible treatment for GERD. The aim was to increase GYP and decrease GYV by constructing a new mucosal flap valve. Thus, it may increase the competence of the antireflux barrier and be effective in controlling the symptoms of GERD.

Due to less invasion, several endoscopic treatments of GERD have been investigated [18], such as collagen or polytetrafluoroethylene injection, laser scarring [19, 20], radiofrequency delivery (RFe) [21], and transoral incisionless fundoplication (TIF) [22]. The disadvantages of these endoscopic techniques included short-term effectiveness, increasing reflux and ulcer, and just tested ex vivo. RFe, for instance, is now widely used in modulating reflux. A study which focused on its complication showed that it may lead to increase reflux and direct superficial burn and ulcerative esophagitis [21]. TIF is another popular endoscopic treatment for GERD. However, an early study on TIF showed lower esophageal acid exposure was reduced in 61% and normalized in only 37% [23].

Inoue et al. first reported the clinical series of ARMS for GERD with no sliding hernia and showed excellent short- and midterm control of GERD [16]. Their two cases underwent repeat endoscopic dilation due to the initial circumferential resection that is too tight. They did not evaluate which the range of ARMS produces the best results. Moreover, the key issue in this procedure is the range of mucosal reduction. So we tried to contrast the different circumference of mucosal resection. As we know, total circumferential mucosal reduction always causes severe esophageal stenosis. In the present study, contrary to the 1/3 circumference of mucosal resection, 2/3 circumference of mucosal resection was effective. The procedure was relatively safe among 9 pigs. No complication was experienced. The ARMS attempted to make the reflux barrier more resistive by shaping a new mucosal flap valve and a full-thickness partial scar after mucosal dissection. We found that a mucosal flap also was rebuilt by EPMR, which could shorten the procedure time than ESD.

The Angelchik prosthesis has been reported that reducing the yield in response to gastric distension was effective in the treatment of GERD [23, 24]. In our study, 2/3 circumference of mucosal resection was effective in increasing the resistance of the LES to reflux. GYV and GYP have been reported firstly as an assessment of reflux resistance of LES pre- and postfundoplication [25]. The creation of fundoplication in baboons increased the GYP by 200% [25]. The GYP was increased by 75% after RFe treatment [26], and a fibrotic nipple valve resulted in a statistical increase of GYP (+51%) [27]. Moreover, ARMS resulted in a statistically significant difference in GYP and GYV versus controls (*p* = 0.004 and *p* = 0.028), which increase the GYP by 53% and GYV by 72%. This finding suggests an effect of ARMS treatment that is similar to RFe treatment for the GYP and GYV. We think the GYP and GYV were significantly changed because the diameter of the lumen changed and a new mucosal flap valve formation occurred. But it also requires clinical trials with larger sample size to assess the antireflux effect of ARMS. Triadafilopoulos et al. [28] found that the total wall thickness and muscle thickness of the LES were significantly thicker after RFe corroborated by histopathological evaluation. In our study, the width of the cardia decreased after ARMS has been demonstrated by the use of barium radiography. As a result of this study, thickening of the mucosa may reduce the compliance of GEJ, shape a new mucosal flap valve, and contribute to ARMS in the treatment of GERD.

There are some limitations of this study. First, an animal model was used in this study. Second, the sample size was small. Third, intrinsic LES pressure was not analyzed. We tried to measure the LESP, but the swines cannot cooperate with swallowing, so we failed to do it. However, a previous study reported that even though the patient's symptom was improved, LES pressure was not increased significantly after the endoscopic fundoplication [29, 30]. Future clinical trials with larger sample size are required to assess the antireflux effect of ARMS.

## 5. Conclusion

Our study here continues to raise the potential antireflux effect of ARMS in GERD treatment. We also recommend the 2/3 circumference resection of the esophagus at 3 cm distance from the GEJ. Clinical trials are necessary in order to offer stronger evidence.

## Figures and Tables

**Table 1:** The clinical characteristics, GYP, and GYV of animals.

	Group A	Group B	Group C	*p*
Sex (male : female)	1 : 2	2 : 1	1 : 2	NA
Weight (kg)	35.17 ± 0.76	33.00 ± 3.61	35.50 ± 1.80	0.427
Operation time (min)	9.67 ± 1.53	45.00 ± 8.54	78.67 ± 6.51	0.006
GYP (cmH_2_O)	13.07 ± 2.10	16.2 ± 1.66	24.23 ± 3.43	0.004
GYV (ml)	2200.00 ± 238.96	1796.67 ± 168.03	1586.67 ± 206.48	0.028

## Data Availability

All the data supporting the results were shown in the paper and can be applicable from the corresponding author.
